# Transcriptomic characterisation and genomic glimps into the toxigenic dinoflagellate *Azadinium spinosum*, with emphasis on polykeitde synthase genes

**DOI:** 10.1186/s12864-014-1205-6

**Published:** 2015-01-23

**Authors:** Jan M Meyer, Christian Rödelsperger, Karsten Eichholz, Urban Tillmann, Allan Cembella, Angela McGaughran, Uwe John

**Affiliations:** Ecological Chemistry, Alfred Wegener Institute for Polar and Marine Research, Bremerhaven, Germany; Evolutionary biology, Max Planck Institute for Developmental Biology, Tübingen, Germany; Adenoviridae: Receptors, Trafficking and Vectorology, Institut de Génétique Moléculaire de Montpellier, Montpellier, France

## Abstract

**Background:**

Unicellular dinoflagellates are an important group of primary producers within the marine plankton community. Many of these species are capable of forming harmful algae blooms (HABs) and of producing potent phycotoxins, thereby causing deleterious impacts on their environment and posing a threat to human health. The recently discovered toxigenic dinoflagellate *Azadinium spinosum* is known to produce azaspiracid toxins. These toxins are most likely produced by polyketide synthases (PKS). Recently, PKS I-like transcripts have been identified in a number of dinoflagellate species. Despite the global distribution of *A. spinosum*, little is known about molecular features. In this study, we investigate the genomic and transcriptomic features of *A. spinosum* with a focus on polyketide synthesis and PKS evolution.

**Results:**

We identify orphan and homologous genes by comparing the transcriptome data of *A. spinosum* with a diverse set of 18 other dinoflagellates, five further species out of the Rhizaria Alveolate Stramelopile (RAS)-group, and one representative from the Plantae*.* The number of orphan genes in the analysed dinoflagellate species averaged 27%. In contrast, within the *A. spinosum* transcriptome, we discovered 12,661 orphan transcripts (18%). The dinoflagellates toxins known as azaspiracids (AZAs) are structurally polyethers; we therefore analyse the transcriptome of *A. spinosum* with respect to polyketide synthases (PKSs), the primary biosynthetic enzymes in polyketide synthesis. We find all the genes thought to be potentially essential for polyketide toxin synthesis to be expressed in *A. spinosum*, whose PKS transcripts fall into the dinoflagellate sub-clade in PKS evolution.

**Conclusions:**

Overall, we demonstrate that the number of orphan genes in the *A. spinosum* genome is relatively small compared to other dinoflagellate species. In addition, all PKS domains needed to produce the azaspiracid carbon backbone are present in *A. spinosum*. Our study underscores the extraordinary evolution of such gene clusters and, in particular, supports the proposed structural and functional paradigm for PKS Type I genes in dinoflagellates.

**Electronic supplementary material:**

The online version of this article (doi:10.1186/s12864-014-1205-6) contains supplementary material, which is available to authorized users.

## Background

Marine dinoflagellates are distributed worldwide from polar regions to tropical seas, where they typically constitute a considerable fraction of the plankton and thus affect the global carbon balance. Although dinoflagellates are often considered as phytoplankton because many are photosynthetic primary producers, other species are facultative or obligate heterotrophs. In addition, dinoflagellates are heavily represented among species known to form “harmful algae blooms” (HABs), thereby having strong impacts on ecosystem services and functioning and potentially affecting human health as their toxins are vectored through food webs into seafood [1].

Dinoflagellates comprise a well-supported monophyletic group based on both molecular data and many unique morphological characters [2]. The Dinophyceae belong to the Alveolata, together with the Ciliata and Apicomplexa. Compared to all other eukaryotes, the genome of dinoflagellates is highly unusual with respect to both structure and regulation [3-7]. The nucleus contains chromosomes that are permanently condensed throughout the cell cycle except during DNA replication [8,9], displaying a liquid crystalline state [10]. Dinoflagellate genomes are among the largest known among eukaryotes, ranging in size from 1.5 to 245 Gbp [11]. The number of protein coding genes is relatively high and is proposed to range from 30,000 to 90,000 [12]. In addition to the structural peculiarities, the large genome size, high gene copy numbers, and a high content of repetitive genomic elements have made genome sequencing and assembly for dinoflagellate species a difficult task. Nevertheless, the draft assembly of the dinoflagellate, *Symbiodinium minutum*, which possesses one of the smallest reported dinoflagellate genomes (1.5 Gbp) was recently published [7]. Additional Transcriptomic studies (e.g. [13]) are beginning to focus research towards in-depth analysis of gene content in dinoflagellates.

The dinoflagellate *Azadinium spinosum* [14] is responsible for the production of potent toxins known as azaspiracids (AZAs) [15], which can accumulate in a variety of shellfish species, causing Azaspiracid Poisoning (AZP) – a severe gastrointestinal illness – in human consumers of such contaminated shellfish. Since the first report of AZP in 1995, AZAs have been found in shellfish from many western European countries and also from African, Chilean, and Chinese coastlines [16-20]. The presence of AZAs has been associated (in all confirmed cases) with members of the genus *Azadinium*, which now comprises more than a dozen species [21]. These observations indicate the widespread biogeographical distribution and potentially important ecological and socio-economic role of *Azadinium*.

The AZPs in *Azadinium* are polyether compounds with structural affinities to the polyketides, a complex and diverse class of secondary metabolites. The polyketides comprise not only potent toxins, but also many compounds with key biomedical functions, as antibiotics, insecticides, and immunosuppressive and anti-tumor agents [22]. Polyketides are synthesized by specific enzymes called polyketide synthases (PKS) through a series of condensation and reduction steps of acyl monomers. PKS enzymes are multi-functional complexes consisting of a minimal set of catalytic domains, namely ketoacylsynthase (KS), acyl transferase (AT) and acyl carrier protein (ACP), which are required for function. Three further domains (ketoacylreductases (KR), dehydrases (DH) and enolreductases (ER)) can be optionally present, and when present, these are responsible for the broad variety of polyketide structures found in dinoflagellates [23].

PKS enzymes can be classified into three groups according to structural and functional elements. Type I PKS enzymes are large multifunctional proteins, comprising either one big protein or organized into modules. In Type II PKS the different domains are organized as individual proteins, which form complexes for polyketide synthesis. The monofunctional, homodimeric Type III PKS act directly on acetyl-Coenzyme A during synthesis without requirement for ACP [24].

Type I PKS genes have been found in many prokaryotes and eukaryotes, including numerous fungi and bacteria [22], apicomplexa [25], haptophytes [26-28], chlorophytes [26] and dinoflagellates [5,29,30]. Phylogenetic analysis has revealed the existence of a protistian PKS clade consisting of apicomplexa, haptophytes, chlorophytes and dinoflagellates [30]. Yet these relationships do not reflect the proposed evolution of their source species [26,31] and therefore little is known about the origin and emergence of PKS genes. The widespread phylogenetic and biogeographical distribution of PKS genes among species provides an interesting tool to study evolution.

The genome size of *A. spinosum* has been estimated to be 9 Gbp [32] and therefore falls into the smaller end of the size spectrum among dinoflagellates. With its widespread occurrence, toxic potential and relatively small genome size, *A. spinosum* is an interesting candidate for study in an evolutionary context. Here, we aim to achieve first insights into genome structure and organization of *A. spinosum.* We perform transcriptomic analysis, comparing data of *A. spinosum* with that of 18 other dinoflagellates (The Marine Microbial Eukaryotic Transcriptome Sequencing Project [33]), and three diatom species (the haptophyte *Emiliania* huxleyi, the cryptomonad *Guillardia theta,* and the chlorophyte *Chlamydomonas reinhardtii*) to identify orphan and homologous genes*.* Specifically, we analyse the transcriptome with respect to the enzymes involved in polyketide synthesis (Type I PKS) and thereby confirm that PKS from *A. spinosum* belong to the dinoflagellate Type I KS sub-group. We further show that the *A. spinosum* genome is less complex, based on sequence repeat structures within its noncoding regions, than that of other dinoflagellates with larger genome sizes, but does not deviate in a major way from the structure and organization typical of free-living dinoflagellates.

## Results

### Features of the *Azadinium spinosum* genome

The 454-shotgun sequencing on a GS Junior System yielded 103,860 high quality genomic reads (37 Mb) (GenBank accession number: SRR1576789). The dataset provided substantial insights into the genomic structure and organization of *A. spinosum*, although the total coverage was rather low for such a big genome. The distribution of genomic features in *A. spinosum* based upon sequence analysis with the program RepeatMasker [34] is given in Table 1. This analysis revealed the overall genomic GC-content to be 49.7% which was lower than the transcriptome GC content of 60.3%. About six percent of the assembled *A. spinosum* genome consisted of simple repeats 2-10 bp in length. Of these, repeats (ATG)n, (TTG)n and (CAT)n were the most common, accounting for 0.005% of the total number of bp. Low-complexity repeats, primarily poly-purine/poly-pyrimidine stretches, or regions of extremely high AT or GC content, made up 0.44% of the *A. spinosum* genome and, of these, A-rich repeats were most abundant followed by GA-rich and then G-rich repeats (Table 1).

**Table 1:** **Genomic characteristics of** ***Azadinium spinosum***

**Genomic feature**	**No. of elements**	**Length occupied (bp)**	**(%) sequence analysed**
Small RNA*	163	30369	0.08
Simple repeats	27167	2357444	6.25
Low complexity	1949	164855	0.44
Retrovirus	69	64400	0.17
Protein-coding	860	374136	0.99
No Database hit	-	34748219	92.07
Total		37739423	100

*Small RNA comprises snRNA and scRNA.

Annotation of the genomic sequences with blastx against the Swissprot database using the blast2go tool resulted in a low annotation success of the genomic DNA data. From a total of 103,860 genomic DNA reads, 860 (0.8%) were identified as potential coding sequence. This corresponds to 374,136 bp (0.99% from a total of 37 Mb; Table 1), with an average read length of 472 bp. Combined with a retrovirus content of 0.17%, the protein coding sequence in *A. spinosum* therefore accounts for 1.16% of the total genomic DNA (Table 1).

The ten most common genes found in the identified protein coding sequence are given in Table 2. Almost all of them belong to energy and primary metabolic pathways; exceptions are transcription factors and proteins involved in mRNA processing (Table 2).

**Table 2:** **Ten most common genes found in** ***Azadinium spinosum*** **genomic sequences**

**Function**	**Reads**	**Blast2go annotation**
Energy metabolism	41	uncharacterized mitochondrial protein g00810 like
Energy metabolism	26	cytochrome c oxidase subunit 1*
Energy metabolism	11	cytochrome b complex III*
Energy metabolism	10	pentatricopeptide repeat-containing protein*
Photosynthesis	56	photosystem q protein*
Photosynthesis	29	photosystem i p700 chlorophyll a apoprotein*
Photosynthesis	16	photosystem i p700 chlorophyll a apoprotein a1*
Pyrimidin metabolism	71	deoxyuridine 5 -triphosphate nucleotidohydrolase
Transcription factor	10	similar to copia protein
mRNA processing	10	splicing factor 3a subunit 2

*Indicates annotations from organelle DNA.

### Features of the *Azadinium spinosum* transcriptome

A total of 75,455 contigs were assembled after Illumina RNA sequencing, of which 844 were carrying the complete spliced leader sequence (DCCGUAGCCAUUUUGGCUCAAG, D = U, A or G). From these contigs, 69,956 protein fragments were predicted, of which 28,357 (40%) could be assigned to proteins in the PFAM database with an *e*-value <0.001.

The completeness of the transcriptomic dataset was determined using the CEGMA tool, which represents a database of 458 highly conserved eukaryotic core genes present in a wide range of eukaryotic taxa [35]. In the *A. spinosum* transcriptome data, 430 (94%) of these conserved gene families were detected. Additionally, we searched for the presence of key metabolic pathways enzymes (for glycolysis, tricarboxylic acid [TCA], pentose phosphate and oxidative phosphorylation pathways) in our transcriptomic data. Among all enzymes checked, only hexokinase (for glycolysis) was absent; instead, we found glucokinase, an enzyme capable of performing a similar reaction to the conversion of glucose into glucose-6-phosphate. Further transcripts highly similar to all histone encoding genes (H2A, H2B, H3, H4), histone methyl-transferases and histone deacetylase were present (see Additional file 1). No evidence supporting a weaker expression of these genes was detected (median reads per base: histones 3.1, all other genes 2.9).

**Figure 1:**
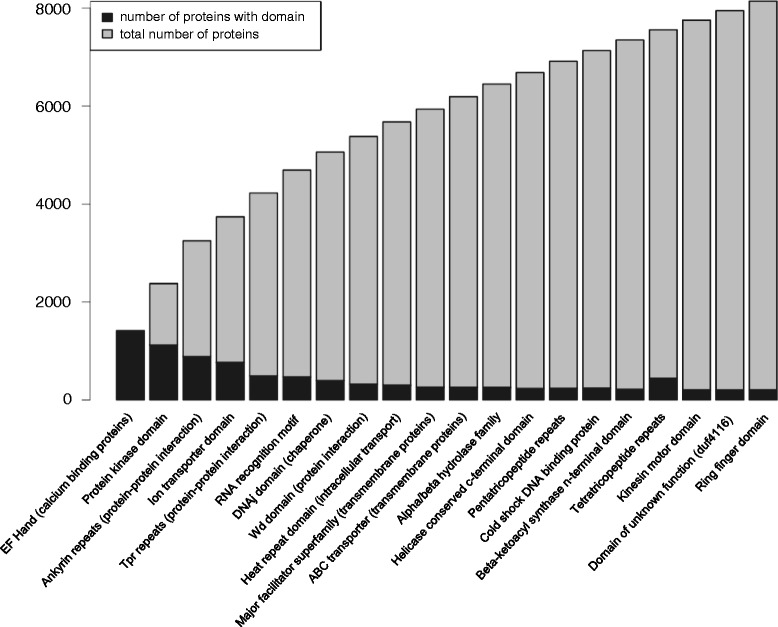
**The 20 most abundant protein domains in the** ***Azadinium spinosum*** **transcriptome.** Black bars denote the number of proteins with a certain domain. Black and grey bars together indicate the total number of proteins for the first *n* domains. The subset and order of domains was iteratively chosen to maximize the total number of proteins (black plus grey bars) for the first *n* domains. The 20 most abundant domains make up 28% of the total proteome (28,357) that could be matched to the PFAM database (e-value 0.001).

Analysis of the translated protein sequences, searched against the PFAM database revealed the most common protein domains in *A. spinosum*. Among these protein domains, protein kinases and, ion transporters – both common domains for protein-protein interaction and ABC-transporters – were found (Figure 1, see Additional file 2).

**Figure 2:**
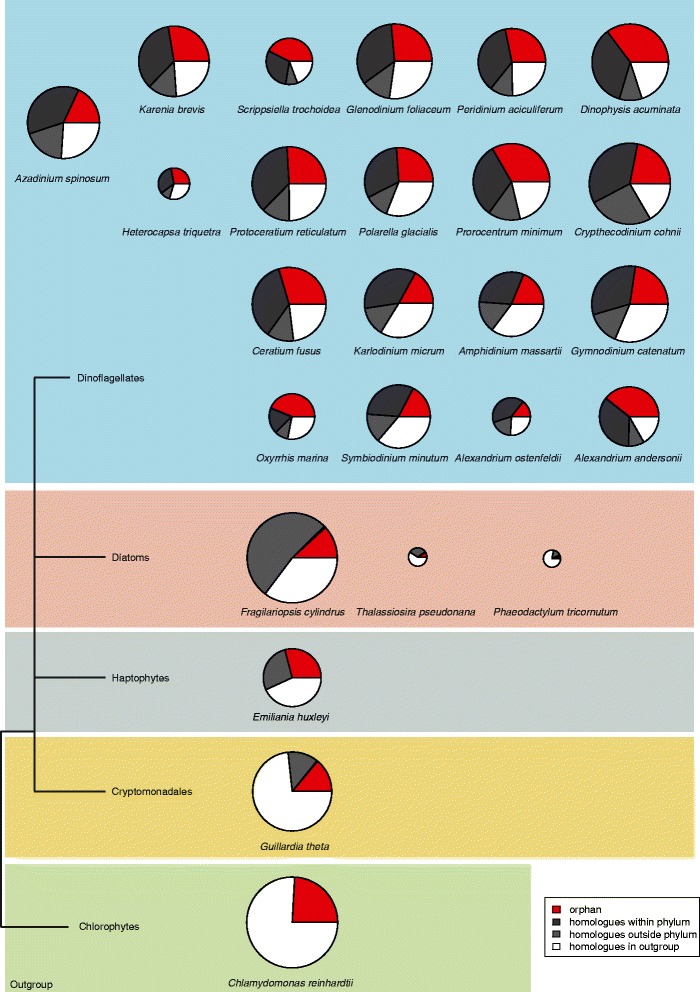
**Transcriptomic data reveals proteins conserved between different dinoflagellates and outgroup species.** Comparison between 19 dinoflagellate species including *A. spinosum*, three diatoms, the haptophyte *Emiliania huxleyi,* and the cryptomonad *Guillardia theta*. The size of the pie charts is proportional to the number of sequences analysed. The colour code indicates the hierarchical classification into homology groups defined by descending evolutionary distance, e.g., if a sequence had BLAST hits in the outgroup species *Chlamydomonas reinhardtii*, it was classified as “homologues in outgroup” irrespective of potential homologues inside the phylum. The schematic phylogeny to the left of the figure represents the relationship between the species analysed. Details about origin and number of sequences analysed can be found in Additional file 3: Table S1.

We compared the predicted protein data of *A. spinosum* (expressed sequence tag, EST data) with EST or whole genome data from 18 other dinoflagellate species (see Additional file 3). For comparison within the Rhizaria Alveolate Stramelopile (RAS) group proteins associated with cellular signal transduction, we conducted a data-base comparison of three diatoms (*Fragilariopsis cylindrus* [36], *Thalassiosira pseudonana* [37] and *Phaeodactylum tricornutum* [38]), the haptophyte *Emiliania huxleyi* [39], and the cryptomonad *Guillardia theta* [40]. In addition, the chlorophyte *Chlamydomonas reinhardtii* [41] served as a representative species of the Plantae. BLAST analysis revealed that 18% of the proteins found in *A. spinosum* represent orphan genes (proteins found in a given species that lack homologues in other species), 37% have homologues in other dinoflagellate species, 19% are shared between *A. spinosum* and the other alveolate groups, and 26% are shared between *A. spinosum* and *C. reinhardtii*. The relative complement of orphan genes varied widely among the tested dinoflagellate species (Figure 2). For example, *A. spinosum*, with 18% orphan genes, together with *Amphidinium massartii*, *Symbiodinium minutum*, *Karlodinium micrum* and *Alexandrium ostenfeldii* (19%, 17%, 17% and 14%, respectively), grouped among the dinoflagellates having the lowest percentage of orphan genes. In contrast, the dinoflagellates *Oxyrrhis marina* and *Scrippsiella trochoidea* were found to have the highest percentages (44% and 42%, respectively) of orphan genes in the comparisons of this study. The average percentage of orphan genes within the dinoflagellates was 27%.

*A. spinosum* produces polyketide toxins, and we therefore paid special attention to the enzymes involved in polyketide biosynthesis. We identified a number of proteins putatively involved in this pathway, by both BLAST analysis and by accessing the KEGG-database. Among these proteins, KS, KR, AT, ACP, ACPS, TE and MT were identified, whereas ER proteins were absent from our dataset (Table 3).

**Table 3:** **Assembled** ***Azadinium spinosum*** **transcripts putatively involved in polyketide synthesis, identified by BLAST analysis against the KEGG database**

**KEGG database hit (p-value < 0.001)**	**Frequency in reads**
Polyketide synthase (PKS)	22
Ketoacyl synthase (KS)	157
Ketoreductase (KR)	25
Acyltransferase (AT)	41
Acyl carrier protein (ACP)	27
Acyl carrier protein synthase (ACPS)	2
Thioesterase (TE)	19
Enoylreductase (ER)	-
Methyltransferase (MT)	200

Next, we assembled six putative PKS mRNA sequences out of the reads matching PKS sequences (GenBank Accession numbers: KM588916-KM588921; Table 4), of which four were complete and two were partial (splice variants). In five of the six cases, BLASTx analysis revealed high similarity to those of another dinoflagellate, *A. ostenfeldii*, for which three sequences exist in GenBank (Ac0019, Ac0038 and 10-x_J14). The remaining sequence was found to be most similar to another dinoflagellate sequence, *Heterocapsa triquetra*; strain HTE5908 (Table 4).

**Table 4:** **Sequence properties of the six ketoacyl synthases (KS) transcripts identified in** ***Azadinium spinosum***

**Sequence ID**	**Length (bp)**	**ORF**	**Amino acids**	**Best BlastX hit**	**Similarity (%)**	**Accession number**
AS3D901	2909	123-2480	785	Ac0019	59	AFW98411
AS3D902	2976	61-2901	946	Ac0019	65	AFW98411
AS3D903	2714	78-2591	837	10-x_J14	58	AFW98413
AS3D904	3071	164-2989	941	Ac0038	71	AFW98412
AS3D905	2902	87-2300	737	Ac0038	68	AFW98412
AS3D906	3009	42-2888	948	HTE5908	61	AFW98414

**Figure 3:**
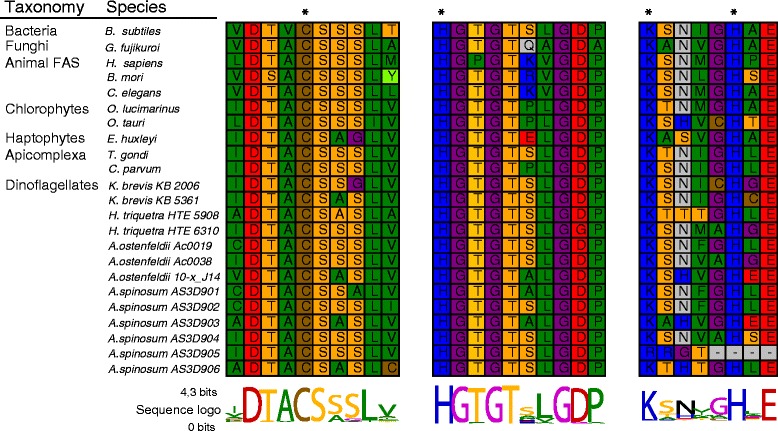
**Amino acids of the ketoacyl synthase (KS) active site motif conserved throughout different evolutionary sub-groups.** The analysis focuses on the protistan Type I PKS in chlorophytes, haptophytes, Apicomplexa and dinoflagellates. The height of the sequence logo given below the alignment represents the degree of conservation. The asterisks indicate the conserved amino acids required for catalytic activity, which are present in all *A. spinosum* sequences (AS3D901-06) except the probably incomplete AS3D905 sequence.

Each of these six transcripts was also analysed *in silico*, yielding theoretical protein sizes of approximately 100 KDa after translation for each transcript. Additionally, the presence of a single ketide synthase (KS) domain in each individual transcript was supported by comparing these transcripts against the PFAM database. This analysis indicated the presence of a single ketide synthase (KS) domain in all transcripts, confirming that they are all PKS-related sequences. All transcripts except AS3D905 contained a DTACSS-motif which includes the conserved amino acids (aa) cysteine, histidine and lysine (Figure 3). The presence of these conserved features supports catalytic activity in these transcripts, as they are known to enable protein function [42] in different phylogenetic lineages (e.g., chlorophytes, haptophytes, apicomplexa and dinoflagellates).

**Figure 4:**
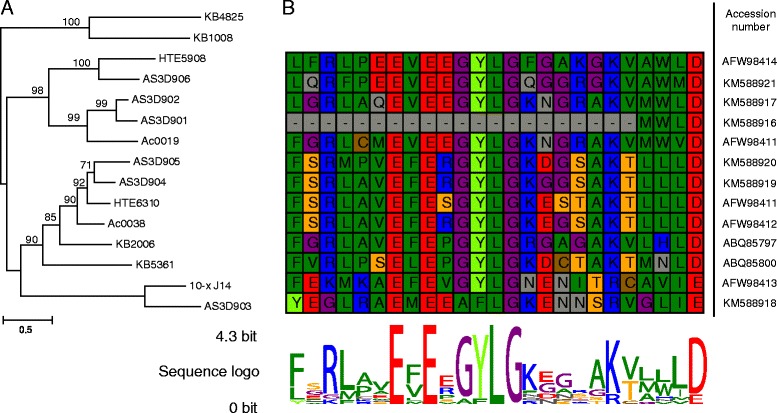
**Phylogeny and multiple alignment of the truncated conserved N-terminal region of the dinoflagellate KS. (A)** Maximum likelihood dendrogram of the N-terminus computed with 1,000 bootstrap replicates. Bootstrap support is indicated on the dendrogram branches; **(B)** Multiple alignment of the conserved N-terminal region. The height of the sequence logo shows the degree of conservation. Each line of the alignment corresponds to the dinoflagellate strain shown in the dendrogram. Outgroup sequences are not shown because they are lacking the N-terminal motif.

In *A. spinosum*, the mean putative protein length among the six identified PKS transcripts was estimated to be 865 ± 84 aa (± s.e.m.). The average protein consisted of an N-terminal region (330 ± 64 aa), a central KS domain with an estimated size of 388 ± 29 aa, and a C-terminal region (147 ± 29 aa). A conserved motif was found in the N-terminal region of all transcripts (Figure 4), which did not yield any database hits via BLAST and PFAM domain search algorithms. Nevertheless, the motif showed sequence similarity to *K. brevis*, *A. ostenfeldii* and *H. triqueta* sequences in the database. This sequence similarity varied from 28 to 51% between *K. brevis* and *A. spinosum*, whereas the maximum sequence similarity between *A. spinosum* and *H. triqueta* (HTE 6310), and between *A. spinosum* and *A. ostenfeldii* (Ac0038) sequences was 64% and 62%, respectively.

### *A. spinosum* PKS transcripts are type I ‘Protistan’

**Figure 5:**
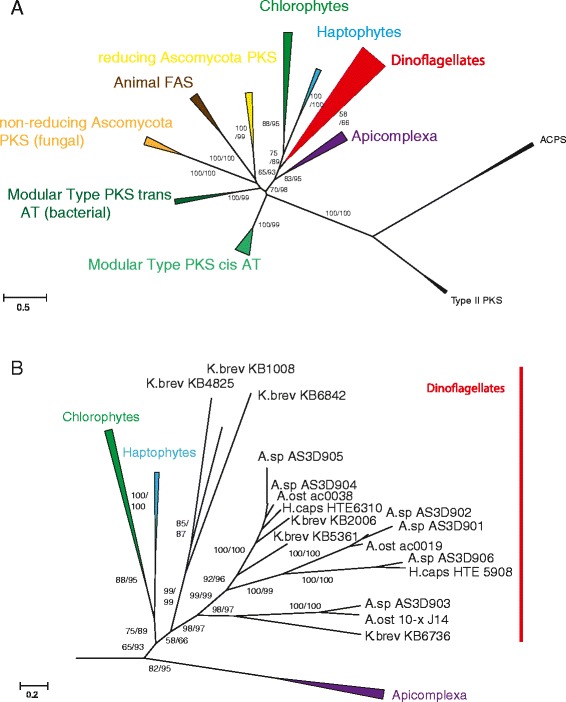
**Phylogenetic tree of Type I and Type II KS domains from prokaryotic and eukaryotic PKS and fatty acid synthase (FAS).** Type I and Type II KS domains from 60 taxa analysed by maximum likelihood approach. Type II PKS and acyl carrier protein synthases (ACPS) were assigned as outgroups. Approximate likelihood fraction (aLRT) and bootstrap values (BT) ≥ 50% are displayed on appropriate branches as aLRT/BT. **(A)** The dinoflagellate KS sequences form a well-supported group within the protistan Type I FAS/PKS clade, consistent with previous topology estimates; **(B)** The dinoflagellate KS group splits into two distinct clades; one contains sequences from different species, the other clade consists exclusively of *K. brevis* sequences.

A phylogenetic maximum likelihood approach yielded insights into relationships of PKS sequences from *A. spinosum* in the current study compared with additional PKS sequences from other dinoflagellates, with an acyl carrier protein synthase (ACPS) sequence as out-group [30]. The resulting overall tree topology was consistent with previously known basic topology for dinoflagellates (Figure 5A). Both ACPS and Type II PKS sequences grouped consistently as out-group taxa with 100% approximate likelihood ratio (aLRT) and bootstrap support (BT). The transcripts in the current study grouped with strong support alongside Type I PKS sequences, indicating that they are also Type I (Figure 5A).

Support for a ‘protistan’ clade of PKS Type I sequences, consisting of apicomplexa, dinoflagellates, haptophytes, and chlorophyta was strong/moderate within the phylogeny (98% aLRT/70% BT; Figure 5A). Each of these major groups, however, formed a discrete sub-group within the protistan clade. The dinoflagellate clade broke down into two clearly separated sub-clades, one of which included sequences from all dinoflagellate species evaluated in this study (including *A. spinosum*, *A. ostenfeldii*, *H. triqueta* and *K. brevis*). The other dinoflagellate sub-group contained only *K. brevis* KS sequences (Figure 5B).

### N-terminal and C-terminal PKS evolution in *A. spinosum*

The phylogenetic relationship of the PKS N-terminal region among the examined dinoflagellates is shown in a maximum likelihood phylogeny in Figure 4A. The tree reflects the same dinoflagellate resolution found in the KS-based phylogeny (Figure 5B), with one sub-clade comprising only *K. brevis* sequences, and the other consisting of sequences from *A. spinosum*, *A. ostenfeldii*, *H. triqueta* and *K. brevis*. The alignment of the N-terminal region revealed several conserved aa positions, including the highly conserved motive ExExGYLG, which was altered in some of the *A. spinosum* sequences (Figure 4B). Specifically, in the sequence AS3D901, the whole dinoflagellate-specific N-terminal region was missing. For AS3D903, the GYL part of the motive was replaced by aliphatic aa.

In a final phylogenetic analysis focusing on the PKS C-terminal region, the BLAST algorithm identified 25% sequence similarity between *A. spinosum* and *K. brevis* (KB 2006), and 64% similarity between *A. spinosum* and *A. ostenfeldii* (Ac0038). The maximum likelihood phylogeny of the C-terminal region generally agreed with the overall (Figure 5A,B) and N-terminal (Figure 4A) phylogenies. We did not find, however, any conserved motif and bootstrap support for most branches was very weak (see Additional file 3: Figure S2).

### Mono-functional organization of polyketide synthase in *A. spinosum*

Both our *in silico* analyses and recent literature reports suggest a mono-functional organization of the KS domain of PKS in *Azadinium* and other dinoflagellates [30,43], rather than a modular one as in most other phylogenetic groups. Western blot analysis with antibodies targeting the KS domain in protein extracts from *A. spinosum* and two other dinoflagellate species (*A. ostenfeldii* and *H. triquetra*) confirmed this interpretation. The blotting pattern showed a distinct band at the expected size (approximately 100 kDA) for a single KS protein (see Additional file 3: Figure S1).

## Discussion

### Features of *Azadinium spinosum* genome

The current study provides the first genomic characterization of the photosynthetic free-living marine dinoflagellate, *A. spinosum* [14]. The genomic sequence of *A. spinosum* consists of a high number of repeat structures of low complexity, with protein coding sequences representing only a small fraction (0.8%) of the genome. Nevertheless, this fraction of protein coding sequences is higher than the 0.2% of protein coding regions of the genomes of both *A. ostenfeldii* (genome size: 105.3 Gb) and *H. triquetra* (genome size: 18.6–23.6 Gb) [5,44].

The ten most common genes in the genomic data were identified based on read counts, suggesting that genes which are covered by more reads may have more copies in the genome. Dinoflagellates are known for their large gene families with sometimes high gene copy numbers [12]. However, our analysis did not allow us to differentiate whether the reads we identified are derived from multiple copies of the same gene within the genome or from different genes from the same gene family.

A minor fraction (0.17%) of the *A. spinosum* sequenced data was assigned to be of retroviral origin. This includes DNA transposons and retrotransposons, which have been found to a similar extent (0.15%) in the *A. ostenfeldii* genome [5]. In the genome of *S. minutum* a slightly higher amount of DNA transposons and retrotransposons (0.5% and 1.1%, respectively) were found. Large tandem repeats, which account for at least 58% of the *A. ostenfeldii* genome [5], were not found in *A. spinosum* and account for 4.6% of the comparatively smaller *S. minutum* genome [7]. Tandem repeats may play a role in chromatin packing and genome organization, similar to what is known for telomere regions [45], thus they may play an important role in the organization of the >50 Gbp genome of *A. ostenfeldii*. Meanwhile, their absence in *A. spinosum* might explain the substantially smaller and less complex genome of this species [32].

### Features of the *A. spinosum* transcriptome

The generated EST data of *A. spinosum* represents a high coverage of the transcriptome. Among all identified transcripts, 844 carried the complete splice leader sequence. Almost all eukaryotic core genes (94% in CEGMA analyses) and the essential enzymes from the key metabolic pathways were found. The difference in GC-content we observed between genome and transcriptome is comparable to what was found previously in *S. minutum* [7].

Unsurprisingly, protein kinase, DNA binding, cytoskeleton proteins, ion channels, and ABC transporter domains were identified among the most abundant protein domains. These domains play important roles in maintaining essential functions in unicellular organisms, e.g., for signaling pathways, gene expression, cellular organization, and homeostasis of ions and small molecules. These findings are similar to published datasets from other dinoflagellates, however in our analysis the number of domains involved in metabolism seems to be less dominant within the top 20 domains [4,5].

As an interesting exception, hexokinase, the key enzyme of glycolysis, was absent from the *A. spinosum* transcriptome. The absence of the hexokinase is an indication that enzymatic activity might have been replaced by another enzyme – in this case, glycokinase - capable of performing similar enzymatic reactions. This is supported by the fact that the reaction catalyzed by both enzymes is essential for energy metabolism. A similar finding was also observed in another study [46]. This could hint towards a general phenomenon in dinoflagellates that should be investigated further, because it suggests a major difference in a basic metabolic pathway.

Additional dinoflagellate genome peculiarities include permanently condensed chromosomes and a low quantity of histones present in the chromatin. Histones have historically been thought to not be expressed or even exist in dinoflagellates [47-49], and the existence of orthologues for all eukaryotic histones has been confirmed only recently [50,51]. Here, we found that sequences potentially encoding proteins of all histone core-units and histone-modifying enzymes are present in *A. spinosum*. We could detect no evidence for a weaker expression of these transcripts in *A. spinosum* compared to transcripts of all other genes. In contrast to our finding in the transcriptome of the dinoflagellate *Lingulodinium* histon mRNAs were found in very low abundance [50], suggesting differences in abundance of histones between dinoflagellate species.

Approximately 40% of the predicted proteins of *A. spinosum* could not be matched to the PFAM database. This is in line with previous findings in dinoflagellates and might be an indication for novel pathways expressed in dinoflagellates [5,52-55]. On average a quarter of the genes found in the dinoflagellate species studied here had no recognizable homologues in other dinoflagellates genera. The estimated number of these ‘orphan’ genes totaled 27% of all genes for the dinoflagellates we studied; this is within the range for other well-studied phyla (e.g. bacteria [56], and nematodes [57]), for which 2-50% of identified genes are orphans.

Different evolutionary mechanisms can lead to the formation of orphan genes. For example, duplication can free gene copies from evolutionary constraints - while the original function is retained in the original gene, the duplicated gene copy can possess new protein functions [58]. An additional source for orphan genes is horizontal gene transfer from other organisms, which has been shown to have the potential to be a significant source of genetic innovation in dinoflagellates [59].

Taken together, a rather unique set of genes may be characteristic for species within the dinoflagellates, potentially reflecting their complex life cycle and nutrition because most are mixo- or heterotrophs and only a few are obligate photoautotrophs [60-62].

### Function and evolution of PKS

All polyketide biosynthetic enzyme functions needed to synthesize the azaspiracid carbon backbone are present in *A. spinosum* (Table 3). This is to our knowledge the first dinoflagellate dataset where all genes needed for polyketide synthesis were identified. Whether these PKS-related sequences are coding, are involved in fatty acid synthesis or toxin production, and/or have another function remains unclear. However, this set of genes opens new avenues to study toxin synthesis in *A. spinosum* and maybe other dinoflagellates also up to now it remains unknown in which cellular compartment toxin synthesis takes place. PKS enzymes have been shown to be expressed in both chloroplast and cytoplasm, but we could not identify signal peptide sequences which may hint towards a specific cellular compartment. However, co-immunoprecipitation studies have shown that PKS functions in *K. brevis* are indeed organized in a mono-modular way, meaning that each domain comprises a single enzyme [29,43].

The KS domain is essential to Type I polyketide synthesis, which is carried out in a stepwise manner. Each KS domain elongates the existing carbon chain by condensation of the acetyl-CoA/malonyl-CoA building blocks, with two carbon atoms normally added in each elongation step. This means that Type I modular PKS must contain at least 20 modules to create the common carbon backbone for the diverse set of identified azaspiracid toxins [15]. We analysed six unique PKS transcripts each containing one KS domain, in detail. Whether or not separate transcripts are used or if the same transcripts are recycled during the polyketide synthesis process remains unclear. In any case, the complete AZA structure could be synthesized with the total number of KS, KR, AT and the other domains identified in our dataset.

Analyses of *A. spinosum* full-length PKS transcripts (Figure 3) and of the corresponding Western blots (Additional file 3: Figure S1) confirms the mono-modular functional Type I PKS structure, which might be a common feature in dinoflagellates [29,30]. It has been proposed that this structure likely diverged from multi-modular PKS around the time of origin of the dinoflagellates [30,43]. The other protistan groups studied here (haptophytes, chlorophytes and apicomplexa) exhibit the multimodular Type I form. The protist KS groups form well-supported monophyletic clades in the current analysis, consistent with earlier findings [26,30].

The flanking regions of KS proteins identified in *A. spinosum* contain less conserved motives than in other organisms, but this sequence feature was also found in other dinoflagellates [30]. In the C-terminal region, in particular, no conserved motif was identified in the alignment, supporting the assumption that this is a relict of the linker sequences of the multi-modular PKS Type I. With this linker structure, the single domains could have been separated to allow structural function of the complex enzyme. Adding *A. spinosum* sequences to the existing C-terminal dataset did not significantly alter the overall topology present in the N-terminal dataset, despite weaker phylogenetic support. In contrast, within the N-terminal sequences, a high degree of conservation was found in the alignment, arguing that the N-terminal has another function rather than acts as a linker region. Instead, the N-terminal region might have an enzymatic or structural function, or could act as a protein-protein binding domain. The conserved N-terminal motif identified previously [30] was found in four of our six *A. spinosum* sequences. However, we observed an alteration of the GYLG motif towards AFLG. Alterations of the GYLG motif have been reported from the dinoflagellate, *Gambierdiscus polynesiensis* and also in some *K. brevis* strains where several variants of the GYLG motive were found [63]. The absence or alteration of the conserved N-terminal motif in the remaining two *A. spinosum* sequences (AS3D901 and AS3D903) could be explained in several ways. For example, these copies might have diverged to play a role in another pathway via gene duplication. Alternatively, these differences may lead to alterations in polyketide synthesis. Finally, these two sequences may simply represent non-functional transcripts or pseudogenes. Functional tests are required to elucidate the role of the PKS genes as well as of the C- and N- terminal sequences.

## Conclusions

We characterized the transcriptome of the toxic dinoflagellate *A. spinosum*, revealing that it possesses a low number of orphan genes compared to other tested dinoflagellate species. With respect to toxin synthesis, we detected transcripts of all genes essential for polyketide synthesis in *A. spinosum*. As the number of transcriptomic datasets for different dinoflagellate species increases, deeper insights into the functional differences between toxic and non-toxic variants, even within a species, as well as into mechanisms of HAB formation, will be gained.

## Methods

### Maintenance and harvesting of dinoflagellate cultures

*Azadinium spinosum* cultures of strain 3D9 isolated from the Scottish east coast (57° 3.9′ N; 02°30.2′W) were grown as described in Tillmann *et al*. [14]. In brief, cultures were grown at 20°C at a salinity of 32 PSU. All exponentially growing cultures were then harvested by centrifugation for 15 min at 3200 × g with fixed angle rotor (Eppendorf 5810 R, Eppendorf, Hamburg, Germany).

### Methods for DNA; extraction

Following centrifugation, cell pellets were re-suspended in Tissue and Cell Lysis Buffer (DNeasy Plant kit; Qiagen, Hamburg, Germany) and transferred into a 2 ml cryovial containing acid-washed glass beads (Sigma-Aldrich, Steinheim, Germany). Cell lysis was achieved with a Bio101 FastPrep instrument (Thermo, Savant Illkirch, France) run at maximum speed (6.5 ms^−1^) for 45 s. DNA extraction proceeded according to the DNeasy Plant kit protocol.

### Shotgun sequencing

Prior to sequencing, DNA was sheared and contaminants and small fragments were removed with the Ampure Bead PCR purification system (Invitrogen, Karlsruhe, Germany) following the standard protocol for 454-library preparation and shotgun sequencing (Roche, Mannheim Germany). Sequencing was then carried out on a Roche GS Junior machine (Roche, Mannheim, Germany) following standard protocols.

### Sequencing post-run processing

The associated 454-sequencing software (Roche GS Junior Version 2.8) was used for quality control and contig assembly. All sequencing reads featuring inaccurate key sequences, chimeric sequences, biased nucleotides, or unidentified nucleotides were regarded as low quality reads and were discarded. A total of 103,860 high quality reads, yielding 37,739,423 bp of genomic information, were retained for analysis.

### Sequence analysis of genomic DNA reads

Low overall genomic coverage and a very large genome size meant that assembly of the retained reads was unrealistic. Reads were thus analysed with the program RepeatMasker ver. 4.0.5 [34] to establish the repeat structure of the genome. To determine the potential proportion of protein-coding sequence, annotation of the high quality reads was also performed. The Blast2go algorithm (www.blast2go.org) was applied against the Swissprot database, and only reads with an e-value below 10^−7^ were considered in the analysis.

### Genome size estimation

The genome size of *A. spinosum* was estimated by DNA extraction and subsequent qPCR. The DNA content was calculated using a standard curve. For more details see [32].

### RNA methods; extraction

Cell pellets obtained after centrifugation of exponentially growing cultures were immediately re-suspended in 1 ml of 60°C hot TriReagent (Sigma-Aldrich, Steinheim, Germany) and transferred into a 2 ml cryovials containing acid washed glass beads (Sigma-Aldrich, Steinheim, Germany). As for DNA extraction, cell lysis was achieved with a Bio101 FastPrep instrument (Thermo Savant Illkirch, France) run at maximum speed (6.5 ms^−1^) for 45 s. RNA isolation was then performed as described in [64]. In brief, after thawing the cell lysate on ice, 200 μl of pure chloroform was added to each sample. The sample was vortexed thoroughly and incubated for 10 min at room temperature. The aqueous phase was separated by centrifugation and transferred into a new vial together with 100% isopropanol and incubated at -20°C to precipitate the RNA. The pellet was collected by centrifugation at 4°C, washed with 70% ethanol, air-dried and re-suspended in RNase-free water (Qiagen, Hilden, Germany).

Only RNA samples with high quality (OD 260/280 > 2 and OD260/230 > 1.8), determined with a NanoDrop ND-100 spectrometer (PeqLab, Erlangen, Germany), and high RNA integrity, checked with the Agilent RNA Nano Chip Assay (Agilent, Santa Clara, USA), were used for transcriptome sequencing.

### RNA sequencing

RNA sequencing and post-run processing was performed by The Marine Microbial Eukaryotic Transcriptome Sequencing Project (MMETSP).

### Analysis of transcriptome data

An assembly pipeline from the MMETSP was to assemble high quality raw reads into contigs, which were translated into amino acid sequences and blasted against the PFAM database (PFAM count (e-value < 0.001) file Supplement).

### Homology analysis

We used the NCBI blast suite (version 2.2.28+) to compare the set of 69,956 predicted protein fragments with gene predictions and EST data from the following species: dinoflagellates: *Alexandrium andersonii, Alexandrium ostenfeldii*, *Amphidinium massartii, Ceratium fusus, Crypthecodinium cohnii, Dinophysis acuminate, Glenodinium foliaceum, Gymnodinium catenatum, Heterocapsa triquetra*, *Karenia brevis*, *Karlodinium micrum, Oxyrrhis marina, Peridinium aciculiferum, Polarella glacialis, Prorocentrum minimum, Protoceratium reticulatem, Scrippsiella trochoidea*, *Symbiodinium minutum*; diatoms: *Fragilariopsis cylindrus, Phaeodactylum tricornutum, Thalassiosira pseudonana*; haptophyte: *Emiliania huxleyi*; cryptomonad: *Guillardia theta*; and the chlorophyte *Chlamydomonas reinhardtii*. We computed all pairwise BLAST comparisons (BLASTp, tBLASTx, BLASTx, and tBLASTn, depending on the type of database) at a threshold of e-value < 0.001 and counted the number of sequences with hits in the other species. For the comparison to the CEGMA data set [35], we used the hmmer package (version 3.0) to detect hits (e-value < 0.001) to any of the CEGMA profile HMMs.

### Phylogenetic analyses

Additional amino acid sequences were obtained from the NCBI GenBank and from [30]. A representative dataset of 65 Type I and Type II PKS sequences covering the major clades of prokaryota, fungi, animals, apicomplexa, haptophytes and chlorophytes was used for maximum likelihood phylogenetic analyses. A multi-sequence alignment was created in MEGA 5.2 [65] with the implemented MUSCLE algorithm [66]. A maximum likelihood tree was computed in PhyML 3.0 [67] under assumption of the La-Gascuel amino acid replacement matrix [68]; likelihood ratio tests [69] and 1,000 bootstrap analyses were performed as a measure of the validity of each branch.

### Analyses of the N- and C-terminal region

The N- and C- terminal regions of the dinoflagellate KS sequences were truncated from the KS domain and blasted with tBLASTx against the NCBI and PFAM databases. No significant hits other than to known KS sequences in the database were obtained by BLASTx analysis. Multiple alignment and subsequent phylogenetic trees were calculated as described for the KS domain.

### Western immunoblotting of dinoflagellate PKS

Western blot analysis of protein extracts from *A. ostenfeldii* AOSH 2 and NCH 85, *A. tamarense* Atam5, *H. triquetra* SCCAP strain K-0481, *E. huxleyi*, *Phaeodactylum sp*., *A. spinosum* and *S. trochoidea* was performed according to [30]. In brief, 10 mg of total protein extract was separated on a 10% SDS/polyacrylamide gel and transferred onto polyvinylidene fluoride membranes by Western Blot. Membranes were blocked with 5% skimmed milk in Tris-buffered saline with 0.5% Tween 20 (TBS-T) for 1 h. Primary antibodies (Rabbit anti-*K. brevis* KS KB 2006 1:5000) were diluted in 5% skimmed milk in TBS-T and the membranes were incubated at 4°C overnight. Subsequently, the membranes were washed three times with TBS-T and incubated with the appropriate secondary antibody dilution (Goat anti-rabbit 1:20000 Peroxidase-conjugated) (Sigma-Aldrich, Schnelldorf, Germany) in 5% skimmed milk/TBS-T for 2 h at room temperature.

## Additional files

Additional file 1:
**List of histon related contigs.**
Additional file 2:
**Most common protein domains based on PFAM database search: pfam_count.**
Additional file 3: Table S1. – Origin and number of the sequences used in the orphan analysis (Figure 2). **Figure S1.** – Presence of KS protein in different microalgae. Western blot analysis of protein extracts from *A. ostenfeldii* AOSH 2 and NCH 85, *A. tamarense* Atam5, *Heterocapsa triquetra* SCCAP strain K-0481, *Emiliania huxleyi*, *Phaeodactylum sp*., *Azadinium spinosum* and *Scrippsiella trochoidea* using a polyclonal rabbit anti- *K. brevis* KS antibody. **Figure S2.** – Phylogeny truncated C-terminal region of the dinoflagellate KS. Maximum likelihood dendrogram of the C-terminus computed with 1,000 bootstrap replicates, bootstrap values ≥ 50% are displayed on dendrogram branches.
