# Supplementary material for: Transcriptomic characterisation and genomic glimps into the toxigenic dinoflagellate Azadinium spinosum, with emphasis on polykeitde synthase genes
Source: BMC Genomics. 2015 Jan 23;16(1):27. doi: 10.1186/s12864-014-1205-6 (PMC4316588; doi:10.1186/s12864-014-1205-6)
Supplement: Additional file 3: Table S1. — – Origin and number of the sequences used in the orphan analysis (Figure 2). Figure S1. – Presence of KS protein in different microalgae. Western blot analysis of protein extracts from A. ostenfeldii AOSH 2 and NCH 85, A. tamarense Atam5, Heterocapsa triquetra SCCAP strain K-0481, Emiliania huxleyi, Phaeodactylum sp., Azadinium spinosum and Scrippsiella trochoidea using a polyclonal rabbit anti- K. brevis KS antibody. Figure S2. – Phylogeny truncated C-terminal region of the dinoflagellate KS. Maximum likelihood dendrogram of the C-terminus computed with 1,000 bootstrap replicates, bootstrap values ≥ 50% are displayed on dendrogram branches. [file 12864_2014_1205_MOESM3_ESM.docx]

# Supporting information

### Additional file 3: Table S1 – Origin and number of the sequences used in the orphan analysis (Figure 2)

| **Species** | **Database** | **Analysed sequences** |
| --- | --- | --- |
| *Chlamydomonas reinhardtii* | JGI | 147924 |
| *Thalassiosira pseudonana* | JGI | 2116 |
| *Phaeodactylum tricornutum* | JGI | 1871 |
| *Guillardia theta* | JGI | 93963 |
| *Fragilariopsis cylindrus* | JGI | 143533 |
| *Emiliania huxleyi* | JGI | 35849 |
| *Oxyrrhis marina* | NCBI EST db | 18024 |
| *Karenia brevis* | NCBI EST db | 65266 |
| *Alexandrium ostenfeldii* | NCBI EST db | 10875 |
| *Heterocapsa triquetra* | NCBI EST db | 6814 |
| *Symbiodinium minutum* | OIST MGU | 47014 |
| *Ceratium fusus* | MMETSP | 75328 |
| *Gymnodinium catenatum* | MMETSP | 83508 |
| *Karlodinium micrum* | MMETSP | 60563 |
| *Amphidinium massartii* | MMETSP | 49772 |
| *Polarella glacialis* | MMETSP | 59754 |
| *Protoceratium reticulatum* | MMETSP | 75927 |
| *Prorocentrum minimum* | MMETSP | 84738 |
| *Scrippsiella trochoidea* | MMETSP | 18897 |
| *Glenodinium foliaceum* | MMETSP | 78761 |
| *Peridinium aciculiferum* | MMETSP | 56916 |
| *Crypthecodinium cohnii* | MMETSP | 101854 |
| *Dinophysis acuminate* | MMETSP | 84397 |
| *Alexandrium andersonii* | MMETSP | 38688 |
| *Azadinium spinosum* | MMETSP | 69956 |

JGI (Joint Genome Institute), MMETSP (Marine Microbial Eukaryote Transcriptome Sequencing Project), NCBI EST db ([National Center for Biotechnology Information](http://www.ncbi.nlm.nih.gov) expressed sequence tags database), OIST MGU ([Okinawa Institute of Science and Technology Marine Genomics Unit](http://www.irp.oist.jp/satoh/))

### Additional file 3: Figure S1 – Presence of KS protein in different microalgae

Western blot analysis of protein extracts from *A. ostenfeldii* AOSH 2 and NCH 85, *A. tamarense* Atam5, *Heterocapsa triquetra* SCCAP strain K-0481, *Emiliania huxleyi*, *Phaeodactylum sp*., *Azadinium spinosum* and *Scrippsiella trochoidea* using a polyclonal rabbit anti- *K. brevis* KS antibody. (uploaded with the figures as “Additional_file_5.pdf”)

### Additional file 3: Figure S2 – Phylogeny truncated C-terminal region of the dinoflagellate KS

Maximum likelihood dendrogram of the C-terminus computed with 1,000 bootstrap replicates, bootstrap values ≥ 50% are displayed on dendrogram branches. (uploaded with the figures as “Additional_file_4.pdf”)
